# Synergistic Role of Selenium and Boron in Enhancing Salinity Tolerance and Secondary Metabolite Accumulation in Oregano

**DOI:** 10.3390/biology14080906

**Published:** 2025-07-22

**Authors:** Mahmut Camlica

**Affiliations:** Agriculture Faculty, Field Crops Department, Bolu Abant İzzet Baysal University, 14280 Bolu, Türkiye; mcamlica25@gmail.com

**Keywords:** nutrient, productivity, chemical contents, stress, crude protein, medicinal plant, secondary metabolite, quality property

## Abstract

The present work was performed in greenhouse conditions, and 10 ppm Se, 10 ppm B, and 100 mM salinity treatments were used. The results showed significant variations in the agronomic traits of oregano among the treatments and harvests. The 10 ppm Se×10 ppm B treatment showed improvements over other properties for morphological and yield properties with protein, essential oil, and Zn contents, and B×salinity outperformed other treatments, with variations in branch number enhancement. The analysis revealed that salinity treatment could effectively enhance Na and Ca contents. B treatment significantly improved Mn (12,443.51–18,739.77 ppm), Mg (406.85–632.79 ppm), Fe (61.43–885.06 ppm), Cu (5.02–9.32 ppm), and B (37.67–114.28 ppm) element contents. The highest K content was found from Se treatment. The effects of the Se, B, and salinity treatments showed effectively after second and third harvests of the oregano for the many examined properties. Fresh and dry weight values showed variability between 1.60–6.00 g/plant and 0.54–2.42 g/plant, respectively. Principal coordinate analysis indicated that most of the properties took place in the groups 1 (1st harvests of plant height and protein content, 2nd harvests of protein content and fresh and dry weight, 3rd harvest of fresh weight and total fresh and dry weight values) and 2 (branch number and essential oil contents, Na, and Zn). The heat-map analysis divided into two main clusters as A and B. A2, A4, and A5 treatments took place in the B group. Treatment of 10 ppm Se×10 ppm B showed better values compared to other treatments. Thus, this treatment may be beneficial for oregano cultivation under non-saline conditions.

## Simple Summary

Salinity is regarded as one of the most damaging environmental stresses, severely limiting crop productivity and quality worldwide. To overcome this stress, it is necessary to apply some mineral substances. Thus, this study aimed to evaluate the effect of selenium and boron treatments on the morphological, yield, crude protein, and mineral matter contents of oregano under salinity stress. Significant physiological and biochemical changes were observed across treatments. The treatment of A7 (10 ppm Se×10 ppm B) had the highest total fresh and dry weight values. In addition, the A3 (10 ppm B) treatment gave the maximum Mg, Mn, Fe, and B contents, while the A4 (10 ppm Se) treatment had the highest K and Se contents.

## 1. Introduction

Selenium (Se) and boron (B), known for their roles in stimulating the synthesis of secondary metabolites in plants, are not regarded as essential nutrients for plant development. It enhances both enzymatic and non-enzymatic antioxidant activities. Beyond their roles in biofortification, Se and B elevate the levels of certain bioactive and health-promoting compounds, potentially enhancing plant physiology and metabolic performance [1,2,3,4,5,6]. The concentrations of Se and B in agricultural products were influenced both the total content and the bioavailability of these elements in the soil [7,8,9,10]. Moreover, Se and B enhanced plant tolerance to a variety of stresses, including salinity, drought, cold, and heavy metals [11,12,13,14,15]. Previous research highlighted beneficial impacts of Se and B on seed germination, yield, and the antioxidative capacity of plants, including soybean, potato, roselle, rye, and lettuce [16,17,18,19,20,21,22,23]. In addition, biofortifying plants through foliar and soil applications of Se and B fertilizers are effective strategies for increasing Se and B levels in the edible portions of plant products [16,17,21]. Like other elements, boron occurs in nature at varying concentrations. However, when present in high concentrations in the soil, boron can become toxic to plants [4,5]. Approximately 50% of the world’s arable land is acidic, and low soil pH decreases the availability of several essential minerals, including boron (B), magnesium (Mg), calcium (Ca), and potassium (K) [15]. In addition, B plays a crucial role in cell wall biosynthesis and structural integrity [24,25]. Specifically, B is involved in the formation of borate esters with rhamnogalacturonan (RG-II), which enhances the porosity and elasticity of the cell wall [26,27]. As a result, the interaction between boron (B) and nitrogen (N) affects the absorption and utilization of nitrogen and other nutrients, including phosphorus (P), potassium (K), calcium (Ca), and magnesium (Mg). This interaction has a significant impact on plant growth parameters such as plant height and other properties [28]. In a study conducted under salt-stress conditions, exogenous Se, B, and Se×B enhanced the enzymatic activity of the antioxidant defense system and glyoxalase systems under different levels of salt stress, ultimately alleviated salt-induced oxidative stress, among which Se+B was more effective than a single treatment in *Glycine max* L. [29].

Oregano (*Origanum onites* L.), belonging to the Lamiaceae family, is native to the Mediterranean region [30,31]. It is traditionally used to address gastrointestinal complaints and has various medicinal treatments, including as an analgesic, antiparasitic, antihelminthic, antitussive, expectorant, sedative, and stimulant [31]. The essential oil of oregano is primarily composed of alpha-terpinene, gamma-terpinene, borneol, carvacrol, linalool, p-cymene, and thymol [31,32,33]. Its cultivation has expanded in recent years, especially in the Mediterranean and Aegean regions. In Türkiye, the cultivated area of oregano expanded from 10.486 ha in 2015 to 18.471 ha in 2020 and further to 21.614 ha in 2023. Corresponding production rates were 8.07, 7.74, and 7.17 tons per hectare, respectively [34].

To our knowledge, this study presents the first report on the morphology, yield, essential oil composition, protein levels, and mineral content of oregano. To our knowledge, no detailed studies have investigated the combined effects of Se and B on oregano under salinity stress. We hypothesized that treatments of Se and B reduce the salinity stress more effectively than combined treatments, improving both the yield and quality properties of *O. onites*. Therefore, the primary objectives of this study were to (i) examine the effects of salt stress and selenium (Se) and boron (B) treatments on vegetative growth parameters and nutrient uptake, (ii) evaluate how salt stress and Se treatment influence essential oil production and crude protein content, (iii) test the hypothesis that Se and B treatments can mitigate salt-stress-induced damage, and (iv) investigate the response of oregano (*Origanum onites* L.) to Se and B treatments under both salt-stressed and non-salt-stressed conditions in a pot-based experiment. Optimizing salt-stress management through Se and B treatments enhances the economic value of oregano.

## 2. Materials and Methods

### 2.1. Plant Material

The used oregano (*Origanum onites* L.) seeds in this study were obtained from the Medicinal and Aromatic Plants Department of the Atatürk Horticultural Central Research Institute, Republic of Türkiye Ministry of Agriculture and Forestry.

### 2.2. Experimental Design and Treatments

The pot experiment was conducted at Bolu Abant İzzet Baysal University Faculty of Agriculture according to completely randomized plot trial design with seven treatments, three replications, and five plants per replication in totally twenty-one pots between 2 January and 13 September 2023, grown under greenhouse conditions.

There were seven treatments, including A1 = tap water as a control, A2 = 100 mM salt concentration, A3 = 10 ppm B, A4 = 10 ppm Se, A5 = 10 ppm B×100 mM salt concentration, A6 = 10 ppm Se×100 mM salinity concentration, and A7 = 10 ppm B×10 ppm Se. In September 2023, seeds were sown in plastic pots (400 mm in diameter) containing 3.5 kg of field soil. The analysis showed that tap water properties had 8.34 pH, 204 ppm EC, 56.8 ppm Ca, 3.26 ppm Mg, 13.59 ppm SO_4_^2−^, 54.71 Cl, 0.49 ppm NO_3_^−^, and lower than 0.5 ppm NO_2_^−^ and NH_4_^+^. Pots were placed in a controlled greenhouse condition (22–27 °C and 55–75% humidity). Generally, the seedling days of plants completed between 12 and 22 days. Six seeds were sown in each pot, and after 22 days of seedling, each pot contained five plants, which were then grown under greenhouse conditions.

During the vegetation period, no fertilizers or chemicals were applied to the plants except for irrigation and experimental treatments. Se, B, and salt-stress treatments were applied on 2 February. Se treatment dose of 10 ppm was developed by dissolving Na_2_SeO_4_ (Sigma-Aldrich, Acros Organics, NJ, USA) in distilled water, B treatment dose of 10 ppm was developed by dissolving Disodium Octaborate Tetrahydrate-Na_2_B_8_O_13_·4H_2_O (Etidot-67—Bandırma Boron and Acid Plants Operation Directorate, Ankara, Türkiye), and NaCl (Sigma-Aldrich, USA) treatment of 100 mM was developed by dissolving it in distilled water. The used soil had high organic matter (3.71%), low phosphorus (0.052 ppm), and medium potassium (108.31 ppm), and it also had 1.14% CaCO_3_, 0.04% total soluble salts, a clay loam texture, and a neutral pH (7.56) [35]. Based on the soil analysis, a basal fertilizer consisting of 40 kg/ha ammonium sulfate and 60 kg/ha diammonium phosphate was applied prior to the Se, B, and salinity treatments. The pots were watered three times in a week with 200 mL distilled water according to needed field capacity during the experiment period [36]. When the plants reached the harvesting time, the harvest of plants were performed by hand about 8–10 cm above ground for the regrowth. The first, second, and third harvests were conducted on 24 April, 6 June, and 13 September 2023, respectively.

### 2.3. Determination of Morphology and Yield Values

Plant height and the number of branches per plant was recorded during harvest, and fresh weight values of the above-ground plant part values were calculated after harvest. The harvested plants were then dried at 40 °C in a thermal drying chamber for approximately one week, until their moisture content reached between 10% and 14%. After drying, the dry weight values of plants were weighted by the precision scale.

### 2.4. Essential Oil Content

The essential oil contents (EOCs) of oregano plants grown under Se, B, and salinity conditions were determined. The essential oil analysis was conducted in the clevenger apparatus (Termal Laboratory, İstanbul, Türkiye). Approximately 20 g grounded dry herbs of oregano was put into glass balloon exposed to 10-times pure water and heated 100 °C during 4 h. After, essential oil amount was measured and put into glass vial (1.5). The obtained content was determined in ml. The samples were repeated three times. The results were converted to %*v*/*w*. Essential oil content was calculated as follows:

EOC = (measured essential oil amount × 100)/grounded dry herb sample.

### 2.5. Crude Protein Content Analysis

Approximately 0.5 ± 0.1 g plant samples were taken and grounded. The samples were digested by boiling with 10 mL of concentrated H_2_SO_4_ (Sigma-Aldrich, USA) and a Kjeldahl selenium digestion tablet (catalyst) (Delta Agriculture, Ankara, Türkiye) until the mixture was clear to put into Kjeldahl distillation unit (Velp Scientific, New York, NY, USA). Then, the distilled water was added on the digest to connect for distillation system (Simsek Laborteknik, Ankara, Türkiye). Sodium hydroxide (40%) (Merck, Darmstadt, Germany) was added to solution as 50 mL to steam distilled ammonia. After 7 min distillation, the solution was exposed to 0.1 N HCI (Sigma-Aldrich, USA), which was included methyl red (Carlo Erba, Milano, Italy) and bromocresol green (Central Drug House Ltd., New Delhi, India) indicators. The titration was continued until it turned into the color change from blue to pink. The calculation of crude protein content was performed comparing to control (without plant sample), and total nitrogen was calculated as % as following formula:

TN (%) = ((S1 − S0) × N × F × Mevn)/Ws

The terms are as follows: TN: total nitrogen; S1: amount of HCl solution used in titration (mL); S0: amount of HCl solution used in blind trial titration (mL); N: normality of HCl solution used in titration (0.1 N); F: factor of 0.1 N HCl solution; Mevn: milliequivalent weight of nitrogen (0.014); and Ws: sample amount (g).

After determination of TN (%), the protein content was calculated by multiplying by 6.25 (F: protein conversion factor).

### 2.6. Determination of Mineral Matter

Mineral matter analysis of the oregano samples was performed using a Perkin Elmer Optima 2100 DV ICP-OES (inductively coupled plasma optical emission spectrometry) device (iCAP 6000 Dual View, Thermo Scientific, Cambridge, UK), with each measurement performed in triplicate. For each sample, 0.3 g was weighed into Teflon digestion vessels, followed by the addition of 5 mL of 65% (*m*/*v*) HNO_3_ (Sigma-Aldrich, USA) and 2 mL of 35% (*m*/*v*) H_2_O_2_ (Sigma-Aldrich, USA). Microwave digestion was carried out according to the manufacturer’s recommended procedure with the following conditions: (i) 145 °C for 5 min, (ii) 200 °C for 10 min, and (iii) 50 °C for 10 min. After cooling, the resulting colorless solutions were quantitatively transferred to 25 mL volumetric flasks and diluted to volume with deionized water. The total concentrations of Ca, Fe, Mg, P, Zn, Cu, and Na were determined using ICP-OES. Results were reported in parts per million (ppm).

### 2.7. Data Analysis

The obtained results were evaluated using analysis of variance (ANOVA) based on a completely randomized plot trial design, and mean comparisons were performed using the least significant difference (LSD) test at a significance level of *p* < 0.05. Principal coordinate analysis (PCA) was performed according to the mean values of the examined parameters. JMP 14 software was used as a statistical analysis program. Heat-map analysis was conducted using the ClustVis 2.0. program.

## 3. Results

### 3.1. Plant Growth Parameters

Table 1 showed the effects of boron, selenium, and salinity treatments on plant height values in oregano plants. No significant difference was found in plant height among the treatments in the first harvest (*p* < 0.05). The results showed that plant height values ranged from 33.23 cm to 41.53 cm in the first harvest. In the second harvest, significant differences were found among the treatments, with plant height ranging from 17.70 cm to 24.40 cm. The A7, A3, and A1 treatments had the highest plant height, and the A2 treatments had the lowest plant height. In the third harvest, significant difference was found in plant height values among the treatments. The plant height values changed between 14.10 and 17.27 cm, and most treatments resulted in similar plant height values, except the A3 treatment in the third harvest (*p* < 0.05, Table 1). When compared to control conditions, the A3 treatment revealed a substantial increase of 12.88%, and the A4 treatment showed a decrease of 3.27% in plant height values of the oregano in the third harvest (*p* < 0.05).

The A3 treatment significantly increased the mean plant height in the second and third harvests compared to the other treatments. The highest of the plant heights with an average 26.48 cm was observed after the treatment of A7 (Table 1). In this experiment, the branch number was affected by the harvest and treatments. Statistical differences were found among the treatments in all harvests with regard to branch number values (*p* < 0.05). Average branch number values in the first, second, and third harvest were 8.01, 12.26, and 15.50 number/plant, respectively. In the first harvest, the A2, A4, and A5 treatments revealed the highest branch number values with 9.00, 8.95, and 8.83 number/plant, respectively. The A6 and A1 treatments had the lowest branch number values with 6.17 and 7.50 per plant, respectively. In the second harvest, the branch number values ranged from 10.13 to 14.33, and the A5 and A7 treatments had the highest values. Applying A6 showed the lowest branch number. Compared to the control treatment, the branch number decreased by 5.33% in the A6 treatment (10.13 number/plant) and increased by 33.93% in the A5 treatment (*p* < 0.05). In the third harvest, the branch number values varied from 12.73 to 17.13. The A5 and A3 treatments had the maximum and minimum branch number values. In addition, the A3 treatment gave rise to a small decrease in the branch number of oregano plants compared to the control group (*p* < 0.05). All treatments significantly increased the branch number (Table 1). The treatment of A4 increased the branch number by 22.12% compared to the A5 treatment.

### 3.2. Fresh and Dry Herb Weights

Table 2 presents the individual and combined effects of Se and B on the fresh and dry weight parameters of oregano plants. No significant differences were observed in the fresh herb weight during the first, third, and total harvests as well as in the dry herb weight of the third harvest (*p* < 0.05, Table 2).

In the first harvest, the fresh herb weights changed between 4.14 and 6.00 g/plant with an average 5.19 g/plant. Statistical differences were observed among treatments in the second harvest regarding fresh herb weight with the treatments. The statistically significant increase (*p* < 0.05) especially demonstrates the efficacy of the A7 treatment for the second harvest. The fresh herb weight ranged from 1.60 to 3.36 g/plant at different treatments, and the highest value was obtained from the A7 treatment in the second harvest. The salinity treatment (A2) resulted in a slight increment (26.26%) in the second harvest of fresh weight relative to the control (A1) treatment (*p* < 0.05, Table 2). When compared to the control (A1) condition, Se and B treatments alone and their combinations with salinity showed substantial increases, ranging from 40.00% to 110% in the fresh herb weight of oregano. All Se treatments were notably higher than all boron treatments. In the third harvest, there was no statistical differences among the treatment doses when the fresh herb weight was recorded by treatments (*p* < 0.05, Table 2). The fresh herb weights ranged from 2.83 g/plant to 3.10 g/plant at different treatments (Table 2). Total fresh weight values ranged from 9.06 g/plant to 12.05 g/plant. Compared to the control treatment, dry herb weight decreased by 5.86% with the salinity treatment (A2), while the other treatments showed increases ranging from 4.73% to 25.18% (Table 2).

The treatment of A7 had the highest total dry weight value, and the first harvest gave the highest dry weight compared the other harvests (Table 2). The dry herb weight in the first harvest changed from 1.51 to 2.42 g/plant, and the A2 treatment showed the lowest value. Compared to the treatments, the A7 treatment had the maximum dry herb weight, followed by the control conditions. Furthermore, the A2 treatment had the lowest value in the first harvest, followed by the A3 and A5 treatments. In the second harvest, the dry herb weights ranged from 0.54 to 1.37 g per plant, with the highest values observed in the A7 and A6 treatments. In contrast to the first harvest, the A1 treatment yielded the lowest dry herb weight, followed by the A2 treatment in the second harvest.

In the third harvest, the dry herb weight of the oregano plants ranged from 1.05 to 1.25 g per plant, with no statistically significant differences among the treatments (*p* < 0.05, Table 2). Treatments A3, A5, A6, and A7 resulted in increases in dry herb weight by 7.53%, 1.27%, 2.25%, and 15.94%, respectively. The A4 treatment showed similar data with the A1 treatment. The total dry herb weight of the oregano plants changed between 3.24 and 5.04 g/plant, and the A7 and A2 treatments showed the highest and lowest total dry herb weights, respectively. Treatments A7 and A6 increased the dry herb weights by 55.56% and 39.51%, respectively (*p* < 0.05, Table 2).

### 3.3. Essential Oil Content (%v/w)

**Figure 1:**
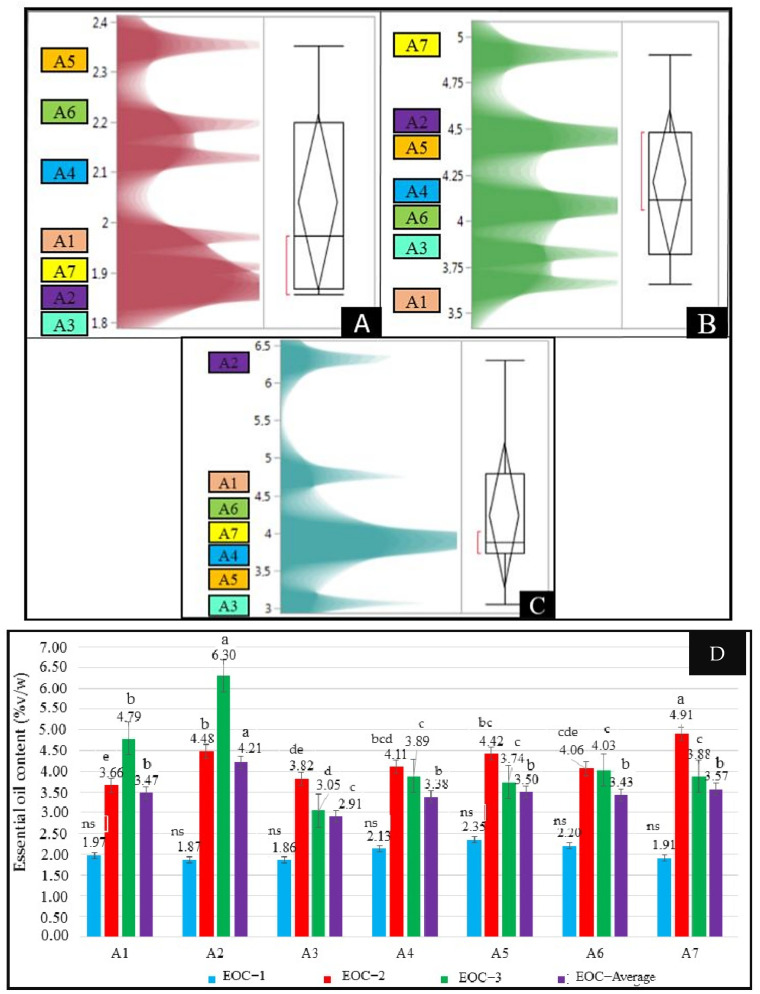
Essential oil contents of the first (**A**), second (**B**), and third harvest (**C**) and the combined values (**D**) of the oregano grown under different treatments. Values with different letters are significantly different at *p* < 0.05 according to the least significant difference (LSD) test. A1: tap water as a control, A2: 100 mM salt concentration, A3: 10 ppm B, A4: 10 ppm Se, A5: 10 ppm B×100 mM salt concentration, A6: 10 ppm Se×100 mM salinity concentration, A7: 10 ppm Se×10 ppm B, ns: not significant, EOC-1: the essential oil content of the first harvest, EOC-2: the essential oil content of the second harvest, EOC-3: the essential oil content of the third harvest, EOC-average: the average essential oil content.

Significant differences were found among the treatments and harvests for essential oil content except in the first harvest (*p* < 0.05, Figure 1). The essential oil content (EOC) changed between 1.86 and 2.35%*v*/*w* with an average of 2.04%*v*/*w* in the first harvest. EOC values ranged from 3.66%*v*/*w* to 4.91%*v*/*w* across the treatments, with the highest recorded in A7 and the lowest in A1 in the second harvest. According to the results of the third harvest, the EOC varied from 3.05%*v*/*w* (for the A3 treatment) to 6.30%*v*/*w* (for the A2 treatment). The A2 treatment showed the maximum EOC, and the A3 treatment had the lowest EOC values. Compared to the first harvest, the EOC increased by 139.88% and 237.33% in the second and third harvest when applying the A2 treatment, respectively. Therefore, a statistically significant increase was observed (*p* < 0.05), indicating a strong effect of the A2 treatment. Similarly, the dry herb yields from the second and third harvests increased by 156.90% and 103.01%, respectively, compared to the first harvest (*p* < 0.05). Therefore, the obtained results from this study revealed that salinity and interactions of Se and B dose treatments positively affect the EOC of oregano. In the average of different harvests, the EOCs of the A1 and A7 treatments were higher than in the other five investigated treatments. In addition, the EOC values of oregano harvested in the second and third harvests were higher than in the first harvest.

### 3.4. Crude Protein Content (%)

**Figure 2:**
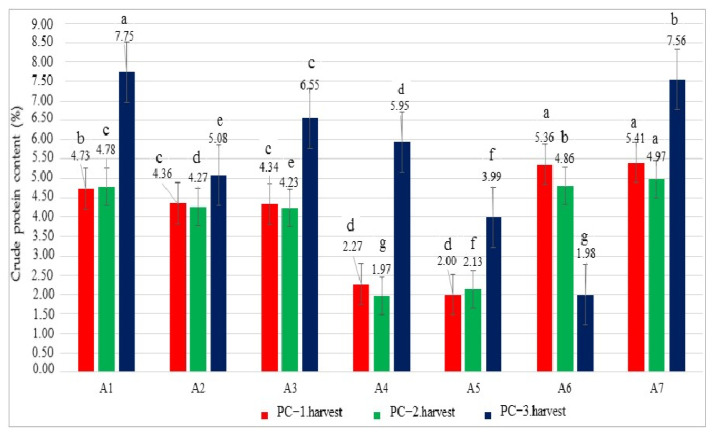
Crude protein content values (%) of the oregano plants under different growing conditions. Values with different letters are significantly different at *p* < 0.05 according to the least significant difference (LSD) test. A1: tap water as a control, A2: 100 mM salt concentration, A3: 10 ppm B, A4: 10 ppm Se, A5: 10 ppm B×100 mM salt concentration, A6: 10 ppm Se×100 mM salinity concentration, A7: 10 ppm Se×10 ppm B. ns: not significant, PC-1.harvest: the protein content of the first harvest, PC-2.harvest: the protein content of the second harvest, PC-3.harvest: the protein content of the third harvest.

In this study, the crude protein contents (CPCs) were calculated for the grown oregano herbs under Se, B, and salinity treatments. The results showed significant differences among the treatments for the CPC and ranged from 2.00% to 5.41% in the first harvest (Figure 2). The highest CPC values were observed in the A7 and A6 treatments, followed by A6 and A1, while the lowest PC was recorded in the A5 and A4 treatments. In the second harvest, the CPC ranged from 1.97% to 4.97%, with the highest and lowest values occurring in the A7 and A4 treatments, respectively. In the third harvest, PC contents showed high variability and varied from 1.98% to 7.75% depending on the treatments. The A1 treatment had the highest CPC, and the A6 treatment had the lowest PC. In addition, the increase was statistically significant (*p* ˂ 0.05), suggesting a robust effect of the A7 treatment in different harvests. Unexpectedly, the protein content under the A1 treatment (control) was higher than in A3, A4, and A7 with increases of 15.48%, 30.25%, and 2.51% (Figure 2, *p* < 0.05). In terms of CPCs, the first and second harvest showed a relationship among the treatments.

### 3.5. Mineral Matter Content

In this experiment, potassium (K) was affected by the treatments. The K contents ranged from 13,339.26 to 18,739.77 ppm, and the average K content for treatments was 15,375.71 ppm (Table 3). Moreover, the A4 and A6 treatments had the highest K contents, and the A5 and A3 treatments had the lowest K contents. It was clearly noted that treatment of Se increased the K content in oregano without salinity.

Magnesium (Mg) contents of the oregano based on the treatments varied from 406.85 to 632.79 ppm with an average of 509.22 ppm. The highest and lowest Mg content were found from the A3 and A7 treatments, respectively. Interestingly, the Mg content decreased the combination of the Se and B treatments as well as the salinity and control conditions.

In the study, sodium (Na) contents changed between 374.22 and 540.75 ppm and showed statistically differences among the treatments. The highest Na values were noted in the treatments of A2 and A5, while the lowest Na contents were found from the A3 and A6 treatments. Compared to the A1 treatment, the A2 and A5 treatments increased the Na content to 4.59% and 12.50%, respectively (*p* < 0.05). Similarly, significant differences were found among the treatments for the calcium (Ca) contents. The Ca contents ranged from 931.51 to 1385.57 ppm, and the highest value was recorded in the A2 treatment. The lowest Ca content was found from the A6 treatment. Compared to the A1 treatment, 15.64, 9.65, and 8.94% increases in Ca contents were observed for the A2, A5, and A3 treatments, respectively. Ca is believed to play a critical role in maintaining cell wall integrity under stress conditions, which may explain the elevated Ca concentrations observed in A2-treated plants.

The results revealed the different impact of treatments on the zinc (Zn) content in oregano. The highest Zn content was recorded in the A6 and A4 treatments among the treatments. The contents of Zn increased with the A6, A4, and A2 treatments, and it decreased with the treatments of A1 and A5. Similarly, the lowest content of Zn was recorded in the treatment of A1.

In this study, the concentration of manganese (Mn) ranged from 15.91 to 74.51 ppm, while iron (Fe) concentrations showed wide variations from 61.43 to 885.06 ppm in the oregano based on the treatments (Table 3, *p* < 0.05). The highest Mn and Fe contents were noted in the treatment of A3, followed by the A2 treatment. On the other hand, the lowest Mn and Fe contents were found from the A7 and A4 treatments, respectively.

Boron (B) and copper (Cu) element values revealed significant differences among the treatments. Table 3 demonstrates the effects of the B and Se treatments on B and Cu contents in oregano under salt-stress condition. According to control treatment, solely Se, B or salinity interaction decreased the contents of B and Cu contents. Moreover, the increase for the B and Cu contents were statistically significant (*p* ˂ 0.05), suggesting a robust effect of A3 treatment (Table 3). While B treatments with salinity conditions significantly increased the contents of B and Cu relative to control conditions, A7 treatment also resulted in a reduction in Cu content. On the other hand, a similar statistical difference was observed in the contents of B and Cu between the A6 treatment. Treatment of B alone significantly improved the contents of B and Cu in oregano plants grown under salinity stress. Finally, it was found that A4 and A6 treatments, at both supplementation levels, did not enhance the content of Cu in oregano plants exposed to salinity stress. The concentrations of both ions in the aerial parts decreased with A2 and A5 treatments. The highest concentrations for Mn and Zn were in the A3 and A6 treatments, respectively. Treatments of the A2 and A3 increased the Mn, Cu and Mg and decreased the fresh weight and essential oil content in the first harvest. Iron and boron contents in the oregano herb increased significantly (*p* < 0.05), demonstrating the strong impact of the A3 treatment. Se content of the treatments ranged from 128.14 ppm to 201.17 ppm, and the A4 and A7 treatments had the highest Se contents. A1 (control) and A2 (100 mM salt) treatments had the minimum Se contents. It was clearly noted that treatment of Se increased the selenium content of the oregano plant grown under Se, B and salinity stress conditions.

### 3.6. Relationship Between Properties

**Figure 3:**
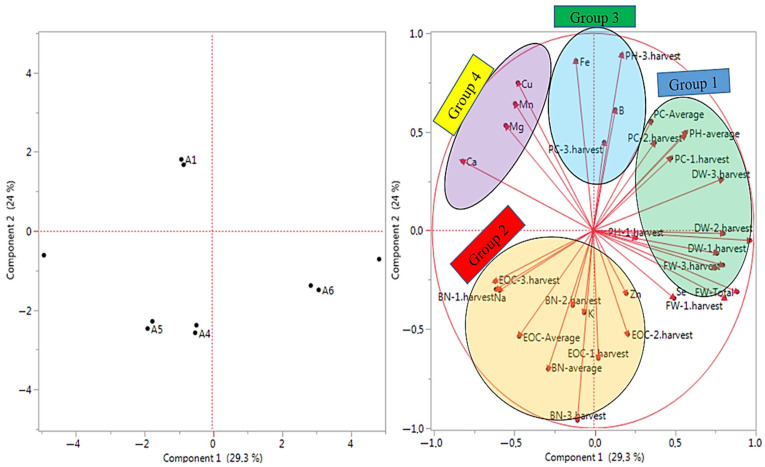
The PCA analysis results of the examined properties based on the growing conditions. A1: tap water as a control, A2: 100 mM salt concentration, A3: 10 ppm B, A4: 10 ppm Se, A5: 10 ppm B×100 mM salt concentration, A6: 10 ppm Se×100 mM salinity concentration, A7: 10 ppm Se×10 ppm B. PH-1.harvest: the plant height of the first harvest, PH-2.harvest: the plant height of the second harvest, PH-3.harvest: the plant height of the third harvest, PH-average: the average plant height, BN-1.harvest: the branch number of the first harvest, BN-2.harvest: the branch number of the second harvest, BN-3.harvest: the branch number of the third harvest, BN-average: the average branch number, FW-1.harvest: the fresh herb weight of the first harvest, FW-2.harvest: the fresh herb weight of the second harvest FW-3.harvest: the fresh herb weight of the third harvest, FW-total: the total fresh herb weight, DW-1.harvest: the dry herb weight of the first harvest, DW-2.harvest: the dry herb weight of the second harvest, DW-3.harvest: the dry herb weight of the third harvest, DW-total: the total dry herb weight, EOC-1: the essential oil content of the first harvest, EOC-2: the essential oil content of the second harvest, EOC-3: the essential oil content of the third harvest, EOC-average: the average essential oil content, PC-1.harvest: the protein content of the first harvest, PC-2.harvest: the protein content of the second harvest, PC-3.harvest: the protein content of the third harvest, PC-average: the average protein content, K: potassium, Mg: magnesium, Na: sodium, Ca: calcium, Zn: zinc, Mn: manganese, Fe: iron, B: boron, Cu: copper, Se: selenium.

The principal component analysis (PCA) analysis was used for examined properties about the quality of data collected from Se and B treatments under salt-stress conditions (Figure 3). The analysis of major components allowed the data to be grouped without relying on a predefined physical model. The PCA was used to identify which observed properties significantly influenced the distribution of the samples. The first major component, PC1, encompassed 30.10% of the variance in the observed data set, whereas the second major component, PC2, explained 24.50% of the variation in the observed data set of the examined properties. A total of 54.60% of variance can be clarified in the observed data set. The evaluated properties in the PCA were distributed across all four quadrants, reflecting groupings based on morphological, yield, and quality traits. The properties of PH-3.harvest, BN-3.harvest, and Fe and Ca contents were placed at extreme positions from the origin in the PCA, whereas the property DW-total was concentrated around the origin on PC1. The PCA results were grouped according to the examined properties to infer relationships among the treatments (Figure 3). As a results, many examined properties took place in group 1, and the least examined properties were found in groups 3 and 4. Therefore, the groups in Figure 3 may have occurred because of the examined properties in the study.

**Figure 4:**
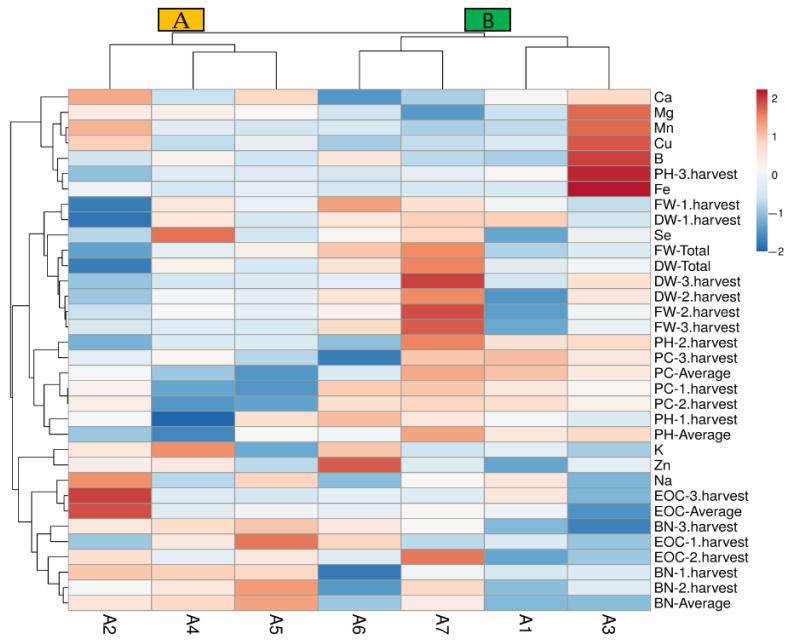
The heat-map analysis results of the studied properties according to the treatments. A1: tap water as a control, A2: 100 mM salt concentration, A3: 10 ppm B, A4: 10 ppm Se, A5: 10 ppm B×100 mM salt concentration, A6: 10 ppm Se×100 mM salinity concentration, A7: 10 ppm Se×10 ppm B. PH-1.harvest: the plant height of the first harvest, PH-2.harvest: the plant height of the second harvest, PH-3.harvest: the plant height of the third harvest, PH-average: the average plant height, BN-1.harvest: the branch number of the first harvest, BN-2.harvest: the branch number of the second harvest, BN-3.harvest: the branch number of the third harvest, BN-average: the average branch number, FW-1.harvest: the fresh herb weight of the first harvest, FW-2.harvest: the fresh herb weight of the second harvest FW-3.harvest: the fresh herb weight of the third harvest, FW-total: the total fresh herb weight, DW-1.harvest: the dry herb weight of the first harvest, DW-2.harvest: the dry herb weight of the second harvest, DW-3.harvest: the dry herb weight of the third harvest, DW-total: the total dry herb weight, EOC-1: the essential oil content of the first harvest, EOC-2: the essential oil content of the second harvest, EOC-3: the essential oil content of the third harvest, EOC-average: the average essential oil content, PC-1.harvest: the protein content of the first harvest, PC-2.harvest: the protein content of the second harvest, PC-3.harvest: the protein content of the third harvest, PC-average: the average protein content, K: potassium, Mg: magnesium, Na: sodium, Ca: calcium, Zn: zinc, Mn: manganese, Fe: iron, B: boron, Cu: copper, Se: selenium.

The data heat-map analysis (Figure 4) identified two main clusters (A and B): the first corresponding to the A2, A4, and A5 treatments (A group) and the second to the other treatments (B group). The A2 treatment was divided from the A4 and A5 treatments depending on the EOC-average, BN-3.harvest, FW-1.harvest, DW-1.harvest, and DW-total properties in group A. The second cluster associated the treatments A1 and A3 in a subgroup and A6 and A7 in another subgroup (Figure 4). In particular, the A1 (control) treatment clustered separately with the A3 treatment, and it may be attributed lower or medium values of PH-2.harvest, BN-1.harvest, and BN-average. The treatments of A4 and A5 separated from the A2 treatment, and the separation may be through the lowest protein contents in the first and second harvest. Similarly, the A6 and A7 treatments clustered in a distinct group from A1 and A3 may indicate synergistic effects of Se and B on higher protein contents of the first and second harvest with lower Fe content (Figure 4).

## 4. Discussion

Selenium (Se) and boron (B) can have both beneficial and harmful effects on the growth, development, yield, and quality of plants depending on their concentrations and the specific plant species involved. The appropriate concentrations of Se and B can help mitigate the effects of various abiotic stresses on plants [25,37,38,39,40]. Hancioglu et al. [41] reported that increasing the salinity of the applied water led to significant decreases in the total fresh weight, total dry weight, dry leaf yield, and total essential oil yield.

In a study examining the effect of salinity stress on daisy (*Matricaria chamomila*) plants, Dadkhah [42] concluded that increased salinity significantly decreases plant height. In this study, the A2 treatment (100 mM salinity concentration) had negative effects on plant height after the first harvest (Table 1). While the plant height in the first harvest of the A2 treatment had a higher value than the A1, A3, and A4 treatments, it was found lower than all treatments in the second and third harvests (Table 1). It was clearly noted that oregano reacted to salinity conditions after the first harvest. Research conducted by Amato et al. [43] reported that plant height values ranged from 68.3 to 78.0 cm by the biostimulant foliar treatments.

In addition to plant height, branch number is an important morphological value for the plants influenced by Se, B, and salinity stress treatments. The results indicate that Se and B supplementations at 10 ppm improve the branch number of oregano plants subjected to the A2 treatment (Table 1). A study reported that among the various treatment combinations with or without Fe, the Se+B treatment alone resulted in the highest relative water content (RWC) and the lowest relative water loss. It also significantly improved growth parameters compared to other treatments [44]. Yu et al. [45] reported that the increasing salinity concentrations decreased the branch number in *Mentha canadensis.* Our results showed that the A2 treatment in the first harvest and the A5 treatment in the second and third harvests had the highest branch number values (Table 1). Therefore, it is thought that salinity stress impacted oregano after the first harvest. However, the treatment of boron (B) might mitigate the effects of salt stress and increase the number of branches.

Previous studies reported that treatments of Se and B had a positive or negative effect on some medicinal plants. Çatav et al. [46] reported that the fresh and dry weights of shoot samples from B1+S1 treatments were 46.4% and 29.3% greater than those from the B1 treatment, respectively, in pepper plants. A study conducted by Yaldiz and Camlica [36] reported that Se treatments significantly improved the dry weight of sage under salt stress. Specifically, it was indicated that the dry weight increased with the combined treatment of 10 ppm of Se and 100 mM/L of NaCl, but it decreased at the highest NaCl concentration (100 mM/L). Similarly, Çamlıca et al. [47] noted that the 5 ppm Se treatment along with low levels of salt stress positively affected the sage dry leaf weight. A study investigating the effects of irrigation water salinity on oregano focused on parameters such as water use, yield, and quality. It was found that increasing salinity levels had a significant negative impact on the plant’s performance. Specifically, the total dry matter weight decreased by 27%, 33%, 44%, and 74% with rising salinity when compared to control conditions. The most substantial reduction was observed at the highest salinity level of 5 dS/m [41]. The findings of this study are consistent with previous reports indicating that the treatments of Se, B, and their combinations enhance yield parameters in plants exposed to salinity stress (Table 2).

Besides morphological and yield values, the EOCs of the plants show wide variations with relation to the geographic origin, extraction analysis, plant phenological stages, growing conditions, and mineral nutrients. Moreover, boron has been reported to induce changes in the EOC and alter metabolite profiles in plants [48,49]. In addition, organic fertilizers release various elements into the soil during their decomposition. These organic fertilizers include macronutrients, micronutrients, and even heavy metals, which have been shown to stimulate the biosynthesis of essential oils in plants [50]. Thus, Hancioglu et al. [41] noted that irrigation water salinity affected the EOC of oregano between 1.58 and 1.84 g/100 g among the different irrigation levels. Amato et al. [43] reported that the essential oil content of the oregano plants ranged from 1.46 to 3.45%*v*/*w* when grown under biostimulant foliar treatments.

In one study, the treatment of 5 ppm Se under salinity stress increased the EOC of sage during the first harvest. Furthermore, increasing Se concentrations in combination with salinity levels further enhanced the EOC [36]. Azimzadeh et al. [51] indicated that the essential oil content increased under low salinity conditions (25 mM NaCl) compared to non-stress conditions. However, as salinity stress intensified with higher concentrations (50 and 100 mM NaCl), the essential oil content decreased. The obtained EOC values (Figure 1) were higher than [41] and similar to [43].

In addition to the EOC, protein content is another critical quality property influenced by mineral matter treatments and stress conditions. Previous studies have reported that salinity stress can have both positive and negative impacts on plants depending on the concentration and type of salt involved. In accordance with this information, protein content significantly decreased in plants treated with 80 mM sodium chloride, whereas it increased in those grown with 160 mM sodium chloride [52]. A key characteristic of salinity stress is the loss of potassium ions from plant roots, leading to a physiological imbalance, as potassium is essential for protein synthesis. Moreover, this loss of potassium can result in diminished plant growth and development [53].

Compared to inorganic forms, organic forms of selenium are more bioavailable and beneficial for humans and animals in the edible parts of plants [54]. Supplementation with Se alleviates salt stress in plants by reducing Na^+^ accumulation in tissues, enhancing Na^+^ compartmentalization, and upregulating genes responsible for Na^+^ and Cl^−^ ion transport. Selenium also contributes to the chelation of toxic ions and boosts the antioxidant defense system, thus protecting plants from oxidative stress. Under salt stress, the excessive production of reactive oxygen species (ROS) is common, and selenium mitigates this by enhancing ROS detoxification mechanisms [55,56,57]. Boron (B) is an essential micronutrient required for plant growth and development, playing a vital role in various physiological processes such as respiration, water and sugar transport, protein synthesis, RNA metabolism, and plant hormone regulation. It is also critical for maintaining the structural integrity of biological membranes [58]. Boron contributes to lignin synthesis and strengthens cell walls, indirectly protecting cell membranes [59]. Under oxidative stress, boron supplementation increases pectin content by 19% and hemicellulose content by 50% in cell walls [60]. Moreover, exogenous boron treatment has been shown to reduce Cl^−^ accumulation in sugar beets under salt stress by enhancing Cl^−^ ion transport [61]. It also decreases lipid peroxidation, evidenced by reduced malondialdehyde (MDA) levels, and lowers hydrogen peroxide (H_2_O_2_) content, thereby enhancing the antioxidant defense system and mitigating oxidative damage in various crops [60,62].

Boron interacts with other mineral nutrients, affecting multiple physiological and biochemical processes [63]. These interactions can be either synergistic or antagonistic, thereby influencing nutrient uptake and utilization. However, the exact mechanisms by which boron deficiency or excess affects mineral dynamics remain not fully understood [20]. Boron is also implicated in nitrogen assimilation, a key process influenced by the interaction between boron and nitrogen, with nitrogen sometimes enhancing or inhibiting boron uptake [64,65].

Under salinity conditions, the reduction in plant dry matter is predominantly associated with disruptions in mineral nutrient uptake and assimilation processes. This finding underscores the critical role of maintaining ionic and nutritional homeostasis to mitigate salinity-induced growth limitations [66]. Therefore, the result of this study revealed the low total fresh and dry weight in A2 applied plants, and it may be attributed to decreasing the mineral matter uptake and utilization.

The A2 treatment (salinity) caused increases in Na^+^ and Ca^2+^ concentrations and slightly decreased K^+^ compared to the other treatments (Table 3). Regarding this situation, Yaldiz et al. [67] reported that salinity treatment has been shown to increase Na^+^ concentrations while reducing K^+^ and Ca^2+^ concentrations in various medicinal and aromatic plants. In this study, it was observed that Ca and Na contents increased whereas K content decreased in the A2-treated plants. This is likely due to competition between K^+^, Ca^2+^, and Na^+^ ions at the root uptake sites. A previous study reported that potassium (K) and calcium (Ca) concentrations of oregano varied from 1.92% to 2.29% and 1.21% to 1.38%, respectively, grown under chemical and organic fertilizers [68]. Under stress conditions, plants with high cytoplasmic salt levels sequester excess Na^+^ in their vacuoles to maintain metabolic functions [68]. In this context, increased Na^+^ uptake in A2-treated plants, likely due to enhanced salt exposure, may be associated with the elevated essential oil content observed in the third harvest. On the other hand, the increased K and Se concentrations observed in A4-treated plants may be attributed to enhanced mineral uptake and accumulation, potentially leading to improved photosynthetic performance. Moreover, selenium treatment may alleviate salinity-induced stress and lipid peroxidation by reducing salt accumulation and promoting the synthesis of photosynthetic pigments. Also, decreased Cu content may be attributed to the antagonistic relationship with A4-applied plants. Also, the Mg, Fe, Zn, Mn, and Cu concentrations were reported between 0.20 and 0.25%, 256.25 and 280.27 mg/kg, 16.16 and 21.00 mg/kg, 19.88 and 44.31 mg/kg, and 9.07 and 13.13 mg/kg, respectively [68]. Magnesium (Mg) plays an important role in the synthesis of protein and energy production, supporting key physiological processes in plants. It is essential for photosynthesis, serving as the central atom in the chlorophyll molecule, which is responsible for capturing light energy. Magnesium also helps activate enzymes involved in various metabolic processes, contributing to overall plant growth and development. Moreover, the high absorption of Mg, Mn, Fe, and Cu elements in A3-treated plants, likely due to the uptake and translocation of other elements, may be associated with the promoted B treatment.

The improved Zn uptake in A6-applied plants may be attributed to enhanced root membrane stability under Se-induced antioxidative protection. Besides the elements mentioned, the Se concentration in oregano was proportional to the amount of Se applied, indicating that the applied selenium was effectively utilized by the oregano compared to control, B, and salt concentration (Table 3). In fact, it has been reported that the treatment of Se fertilizers on the soil or foliar treatment increased the Se content in plants [69,70,71,72]. The findings in this study on Se content in oregano are supported by previous studies. In addition, applied salinity increases the external osmotic pressure and is thought to decrease the amount of B and Se in oregano.

In edible parts of plants, organic forms of selenium are more effective for humans and animals than inorganic forms [54].

This study revealed that the PCA and heat-map analysis showed relationships among the examined properties. Most of the morphological and yield properties especially occurred in the same group. These findings supported the previous study reported by Amato et al. [43], who noted that the PCA analysis (PC1 and PC2) accounted for 75.83% of the total variance combined study of 21 traits that applied biostimulant foliar treatments on oregano. The heat map identified two main clusters where the treatments with biostimulants were separated from the control. In a different study reported by Sarrou et al. [73], PC1 discriminated the Greek oregano accessions with an explained variance of 38.5% while PC2 with 16.2%. Also, combining these data with a biplot analysis revealed that morphometric and agronomic characters contributed to the distinctness of accessions.

## 5. Conclusions

The efficiency of different dose treatments of Se and B in the morphology and yield of oregano as well as their effect in the essential oil, protein, and mineral contents of oregano were evaluated in this study in response to 100 mM salinity treatment. Considering the parameters evaluated, the A7 treatment at a dose of 10 ppm of Se and B and the A2 treatment at a dose of 100 mM salinity are considered promising for the total fresh and dry herb weight of oregano. Mean values of the A2 and A7 treatments promoted a change in the total essential oil and protein content, respectively. The A3 treatments especially increased important mineral matter contents such as Mg, Fe, B.

Se and Se×salinity treatments were responsible for the greater absorption efficiency of the K and Zn and efficiency of the A3 treatment, implying the highest Mg, Mn, Fe, and B contents and accumulation observed in the herb of the oregano. In short, it is recommended to apply 10 ppm Se to oregano plants combined with 10 ppm B to improve the fresh and dry weights of the plants as well as Mn, Mg, Fe, and B compared to other treatments. The used Se and B treatments may offer substantial promise for sustainable agriculture by enhancing salinity stress tolerance in oregano.

## Figures and Tables

**Table 1:** Plant height (cm) and branch number (number/plant) of oregano under different treatments.

Treatments	PH-1. Harvest	PH-2. Harvest	PH-3. Harvest	PH-Average	BN-1. Harvest	BN-2. Harvest	BN-3. Harvest	BN-Average
A1	38.75 ns	22.20 ab	15.30 ab	25.42 ns	7.50 ab	10.70 cd	13.70 ab	10.63 b
A2	38.87	17.70 b	14.10 b	23.56	9.00 a	12.40 abc	16.30 a	12.57 ab
A3	37.64	22.53 ab	17.27 a	25.81	7.62 ab	11.80 bcd	12.73 b	10.72 b
A4	33.23	19.73 ab	14.80 ab	22.59	8.95 a	13.00 ab	16.73 a	12.89 a
A5	40.36	19.67 ab	14.80 ab	24.94	8.83 a	14.33 a	17.13 a	13.43 a
A6	41.53	18.13 b	14.60 b	24.76	6.17 b	10.13 d	16.20 ab	10.83 b
A7	40.10	24.40 a	14.93 ab	26.48	7.98 ab	13.47 ab	15.73 ab	12.39 ab
Average	38.64	20.62	15.11	24.79	8.01	12.26	15.50	11.92
LSD (5%)	14.79	5.55	2.65	5.05	2.41	2.15	3.55	1.96
CV (%)	21.52	15.13	9.86	11.46	16.93	9.86	12.88	9.24

Values within a column followed by different letters are significantly different at *p* < 0.05, according to the least significant difference (LSD) test. A1: tap water as a control, A2: 100 mM salt concentration, A3: 10 ppm B, A4: 10 ppm Se, A5: 10 ppm B×100 mM salt concentration, A6: 10 ppm Se×100 mM salinity concentration, A7: 10 ppm Se×10 ppm B. ns: not significant, PH-1: the plant height of the first harvest, PH-2: the plant height of the second harvest, PH-3: the plant height of the third harvest, BN-1: the branch number of first harvest, BN-2: the branch number of the second harvest, BN-3: the branch number of the third harvest, LSD: least significant difference test, CV: coefficient variation.

**Table 2:** Fresh and dry herb weights (g/plant) of the oregano grown under different treatments.

Treatments	FW-1. Harvest	FW-2. Harvest	FW-3. Harvest	FW-Total	DW-1. Harvest	DW-2. Harvest	DW-3. Harvest	DW-Total
A1	5.19 ns	1.60 b	2.83 ns	9.63 ns	2.42 a	0.54 d	1.08 ns	4.03 ab
A2	4.14	2.02 b	2.90	9.06	1.51 b	0.69 cd	1.05	3.24 b
A3	4.80	2.36 b	2.93	10.08	1.94 ab	1.10 ab	1.16	4.20 ab
A4	5.51	2.39 b	2.91	10.26	2.29 ab	0.96 bc	1.08	4.34 ab
A5	5.11	2.24 b	2.90	10.80	1.97 ab	0.88 bcd	1.09	3.94 ab
A6	6.00	2.48 ab	3.01	11.49	2.29 ab	1.12 ab	1.10	4.52 a
A7	5.58	3.36 a	3.10	12.05	2.42 a	1.37 a	1.25	5.04 a
Average	5.19	2.35	2.94	10.48	2.12	0.95	1.12	4.19
LSD (5%)	2.23	0.91	0.77	3.23	0.82	0.37	0.33	1.14
CV (%)	24.18	21.77	14.63	17.34	21.70	21.66	16.46	15.26

Values within a column followed by different letters are significantly different at *p* < 0.05 according to the least significant difference (LSD) test. A1: tap water as a control, A2: 100 mM salt concentration, A3: 10 ppm B, A4: 10 ppm Se, A5: 10 ppm B×100 mM salt concentration, A6: 10 ppm Se×100 mM salinity concentration, A7: 10 ppm Se×10 ppm B, ns: not significant, FW-1: the fresh weight of the first harvest, FW-2: the fresh weight of the second harvest, FW-3: the fresh weight of the third harvest, DW-1: the dry weight of the first harvest, DW-2: the dry weight of the second harvest, DW-3: the dry weight of the third harvest, LSD: least significant difference test, CV: coefficient variation.

**Table 3:** Mineral matter contents (ppm) of the oregano plants grown under Se, B and salinity conditions.

Treatments	K	Mg	Na	Ca	Zn	Mn	Fe	B	Cu	Se
A1	14,848.15 d	463.67 f	480.64 c	1198.15 d	18.01 e	19.23 f	71.71 e	37.67 f	5.73 d	128.14 f
A2	16,612.65 c	540.34 b	540.75 a	1385.57 a	23.97 b	61.59 b	196.91 b	45.29 d	7.82 b	141.38 e
A3	13,339.26 f	632.79 a	374.22 g	1305.27 c	21.95 c	74.51 a	885.06 a	114.28 a	9.32 a	157.76 d
A4	18,739.77 a	533.58 c	398.12 e	1072.14 e	24.52 b	28.81 c	61.43 g	68.24 c	5.36 e	201.17 a
A5	12,443.51 g	519.09 d	502.69 b	1313.73 b	20.20 d	22.61 e	142.97 c	45.30 d	6.13 c	147.28 e
A6	17,656.67 b	468.20 e	378.69 f	931.51 g	29.27 a	23.84 d	65.66 f	74.84 b	5.02 f	165.16 c
A7	13,989.97 e	406.85 g	457.18 d	1040.99 f	21.49 c	15.91 g	76.65 d	41.31 e	5.36 e	180.64 b
Average	15,375.71	509.22	447.47	1178.19	22.77	35.21	214.34	60.99	6.39	160.22
LSD (5%)	0.67	2.60	0.00	0.00	0.87	0.00	0.87	0.87	0.03	6.57
CV (%)	0.00	0.29	0.00	0.00	2.14	0.00	0.23	0.81	0.26	2.30

Values in a column with different letters are significantly different at *p* < 0.05 according to the least significant difference test. A1: tap water as a control, A2: 100 mM salt concentration, A3: 10 ppm B, A4: 10 ppm Se, A5: 10 ppm B×100 mM salt concentration, A6: 10 ppm Se×100 mM salinity concentration, A7: 10 ppm Se×10 ppm B. LSD: least significant difference test, CV: coefficient variation. K: potassium, Mg: magnesium, Na: sodium, Ca: calcium, Zn: zinc, Mn: manganese, Fe: iron, B: boron, Cu: copper, Se: selenium.

## Data Availability

Data are contained within the article.

## Abbreviations

The following abbreviations are used in this manuscript:

ANOVA	Analysis of variance
BN	Branch number
CPC	Crude protein content
CV	Coefficient variation
DW	Dry herb weight
EOC	Essential oil content
FW	Fresh herb weight
HCI	Hydrochloric acid
ICP OES	Inductively coupled plasma-optical emission spectrometry
LSD	Least significant difference
MDA	Malondialdehyde
Mevn	Milliequivalent weight of nitrogen
mL	Milliliter
mM	Millimolar
ns	Not significant
NaCI	Sodium chlorite
PCA	Principal component analysis
PH	Plant height
ppm	Parts per million
ROS	Reactive oxygen species
RWC	Relative water content
TN	Total nitrogen
